# HMG-box domain stimulation of RAG1/2 cleavage activity is metal ion dependent

**DOI:** 10.1186/1471-2199-9-32

**Published:** 2008-04-01

**Authors:** Aleksei N Kriatchko, Serge Bergeron, Patrick C Swanson

**Affiliations:** 1 Department of Medical Microbiology and Immunology, Creighton University Medical Center, 2500 California Plaza, Omaha, NE, USA

## Abstract

**Background:**

RAG1 and RAG2 initiate V(D)J recombination by assembling a synaptic complex with a pair of antigen receptor gene segments through interactions with their flanking recombination signal sequence (RSS), and then introducing a DNA double-strand break at each RSS, separating it from the adjacent coding segment. While the RAG proteins are sufficient to mediate RSS binding and cleavage *in vitro*, these activities are stimulated by the architectural DNA binding and bending factors HMGB1 and HMGB2. Two previous studies (Bergeron *et al.*, 2005, and Dai *et al.*, 2005) came to different conclusions regarding whether only one of the two DNA binding domains of HMGB1 is sufficient to stimulate RAG-mediated binding and cleavage of naked DNA *in vitro*. Here we test whether this apparent discrepancy is attributed to the choice of divalent metal ion and the concentration of HMGB1 used in the cleavage reaction.

**Results:**

We show here that single HMG-box domains of HMGB1 stimulate RAG-mediated RSS cleavage in a concentration-dependent manner in the presence of Mn^2+^, but not Mg^2+^. Interestingly, the inability of a single HMG-box domain to stimulate RAG-mediated RSS cleavage in Mg^2+ ^is overcome by the addition of partner RSS to promote synapsis. Furthermore, we show that mutant forms of HMGB1 which otherwise fail to stimulate RAG-mediated RSS cleavage in Mg^2+ ^can be substantially rescued when Mg^2+ ^is replaced with Mn^2+^.

**Conclusion:**

The conflicting data published previously in two different laboratories can be substantially explained by the choice of divalent metal ion and abundance of HMGB1 in the cleavage reaction. The observation that single HMG-box domains can promote RAG-mediated 23-RSS cleavage in Mg^2+ ^in the presence, but not absence, of partner RSS suggests that synaptic complex assembly *in vitro *is associated with conformational changes that alter how the RAG and/or HMGB1 proteins bind and bend DNA in a manner that functionally replaces the role of one of the HMG-box domains in RAG-HMGB1 complexes assembled on a single RSS.

## Background

Antigen receptor genes are assembled from component variable (V), diversity (D), and joining (J) gene segments through a site-specific DNA rearrangement process called V(D)J recombination (for reviews, see [1,2]). This process is initiated by two lymphoid cell-specific proteins, called RAG1 and RAG2 (recombination activating genes-1 and -2), which collaborate to bring two gene segments into close proximity by establishing protein-DNA contacts with a conserved recombination signal sequence (RSS) that flanks each gene segment. The RAG proteins subsequently catalyze the formation of a DNA double-strand break at the junction between the coding segment and the RSS through a two-step nick-hairpin mechanism. Each RSS contains a highly conserved heptamer and nonamer element separated by relatively nonconserved spacer DNA that is typically either 12 or 23 bp in length (12-RSS or 23-RSS, respectively). Typically, pairs of gene segments targeted for rearrangement have RSSs containing different lengths of spacer DNA (one 12-RSS and one 23-RSS), a restriction termed the 12/23 rule.

Early biochemical studies of purified RAG proteins established that RAG1 and RAG2 are both necessary and sufficient to support cleavage of isolated RSS oligonucleotide substrates *in vitro *[3]. However, high mobility group proteins that belong to the HMG-box family of architectural DNA binding and bending factors (e.g. HMGB1 or HMGB2) were later found to stimulate RAG binding and cleavage of isolated RSSs (particularly the 23-RSS), and facilitate synapsis and coupled cleavage of RSS pairs according to the 12/23 rule of V(D)J recombination [4,5]. Mammalian HMGB1 and HMGB2 contain tandem homologous DNA binding domains called HMG-box A and B [6,7]. Each domain is about 80 amino acid residues in length and consists of an extended N-terminal strand followed by three alpha helices that fold into an L-shaped structure. A short basic linker connects box B to a C-terminal acidic tail containing about 30 contiguous aspartate and glutamate residues. While both HMG-box domains interact with DNA, they exhibit distinct DNA binding properties: whereas box A prefers to bind structurally distorted DNA, box B lacks this selectively, but can itself induce severe bends into linear DNA, which is a property box A lacks [8-11]. The DNA binding activity and functional properties of the HMG-box domains are strongly influenced by flanking basic and acidic regions of these proteins [11-15].

The distinct biological properties of the various regions in HMGB1/2 led us to speculate that they may play separable roles in promoting the DNA binding and cleavage activities of the RAG proteins. To address this possibility, we previously prepared an extensive panel of truncated and mutant HMGB1 proteins and tested their ability to promote RAG-mediated RSS binding and cleavage *in vitro *[16]. We presented evidence that both single HMG-box domains and full-length HMGB1 could supershift a RAG-RSS complex detected using an electrophoretic mobility shift assay (EMSA). However, whereas association of full-length HMGB1 with the RAG-RSS complex stimulated the RSS cleavage activity of the complex, single HMG-box domains failed to do so. A comparable study published by Dai *et al*. also reported that single HMG-box domain proteins promote RAG-RSS complex formation, but, in apparent contrast to our results, the authors found that single HMG-box domains can stimulate RAG-mediated cleavage in a concentration-dependent manner [17]. We speculated that the conflicting results could be traced to two possible differences in experimental methodology: in our study, RSS cleavage was assessed in a discrete protein-DNA complex using an in-gel cleavage assay in the presence of Mg^2+^; in the study by Dai *et al*., cleavage activity was assessed using a standard *in vitro *cleavage assay in the presence of Mn^2+^. Therefore, we compared the stimulatory effect of varying concentrations of wild-type, truncated, and mutant HMGB1 proteins on RAG-mediated binding and cleavage in the presence of Mg^2+ ^or Mn^2+^. We find that individual HMG-box domains stimulate RAG-mediated cleavage of a single RSS in Mn^2+^, but not Mg^2+^; this observation is consistent with and largely reconciles the conflicting data published by Bergeron *et al*. and Dai *et al*. Interestingly, when these assays are repeated under conditions favouring synapsis (i.e. in the presence of Mg^2+ ^and partner RSS), we find that individual HMG-box domains gain the ability to stimulate RAG-mediated RSS cleavage. However, these distinct outcomes are not attributed to differences in the DNA binding activity of the RAG proteins in Mg^2+ ^and Mn^2+^, as RAG-HMGB1-RSS complex formation is generally comparable using either metal ion as assessed by electrophoretic mobility shift assay.

## Results

### Single HMG-box domains stimulate RAG-mediated *in vitro *23-RSS cleavage in Mn^2+^, and not Mg^2+^, but fail to suppress aberrant nicking by the RAG complex

**Figure 1:**
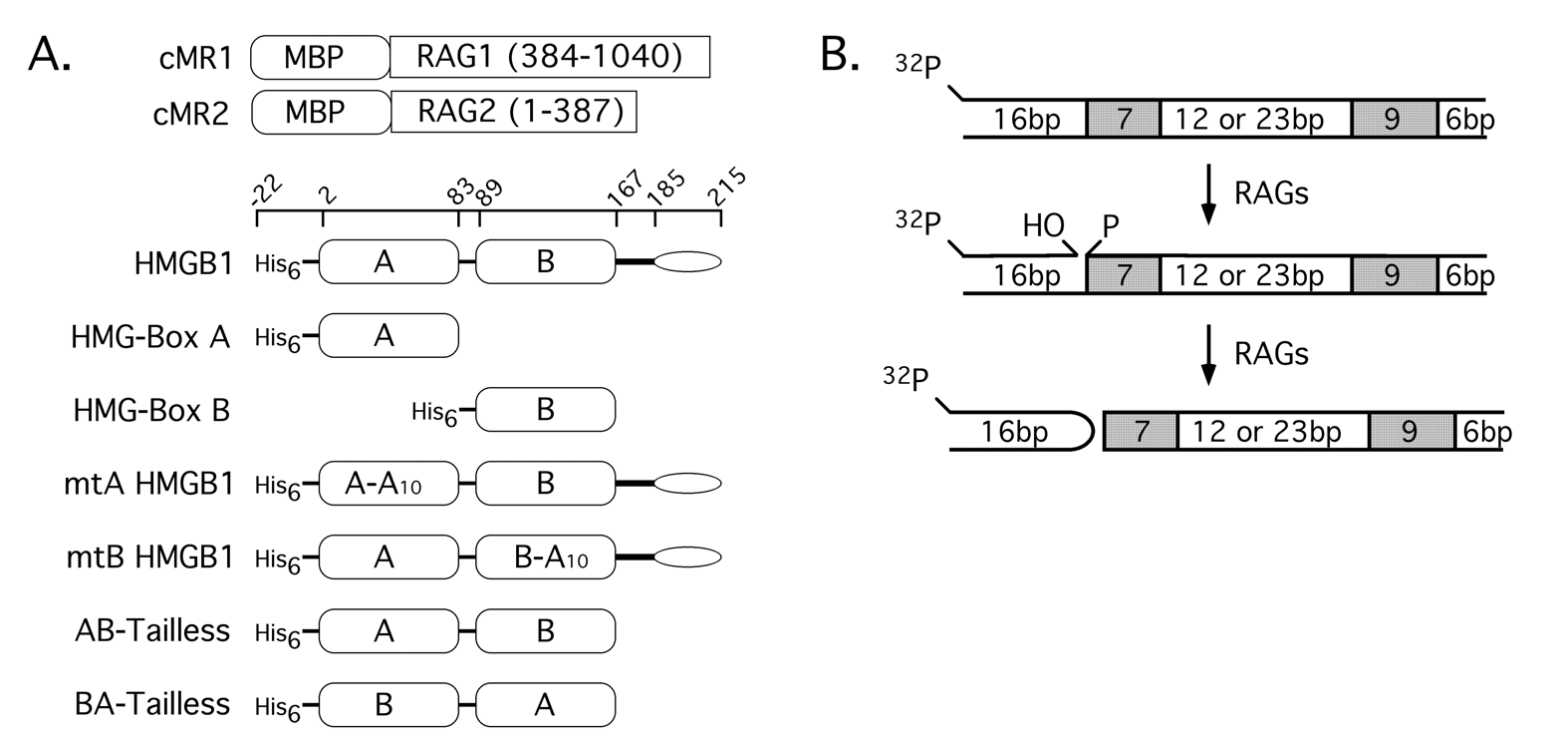
**Proteins and DNA substrates used in this study**. (A) Diagram of proteins used in this study. Maltose binding protein (MBP) tagged forms of truncated RAG1 (384–1040) and RAG2 (1–387) (cMR1 and cMR2, respectively) were coexpressed in 293 cells and purified by amylose affinity chromatography. Full-length, truncated, and mutant forms of recombinant HMGB1 are illustrated below a diagram indicating the residues encompassing the amino-terminal polyhistidine tag (His_6_), the HMG box domains (rectangles), the basic linker (heavy line) and the acidic tail (oval). Mutant forms of full-length HMGB1 contain ten consecutive alanine substitutions (A_10_) in box A (mtA HMGB1, residues 18–27) or box B (mtB HMBG1, residues 102–111). Tailless forms of HMGB1 contain box A and box B in their wild-type configuration (AB Tailless) or are in reverse order (BA Tailless). (B) Diagram of the 23-RSS substrate used in this study. The substrate is radiolabeled on the top strand at the 5'-end (^32^P) and the heptamer and nonamer motifs are shaded. The lengths of the flanking and spacer regions are also indicated. RAG-mediated cleavage proceeds via a two-step mechanism involving top strand nicking at the 5'-end of the heptamer, followed by direct transesterification to liberate a blunt, 5'phosphorylated signal end, and a coding end terminating in a DNA hairpin structure [3].

**Figure 2:**
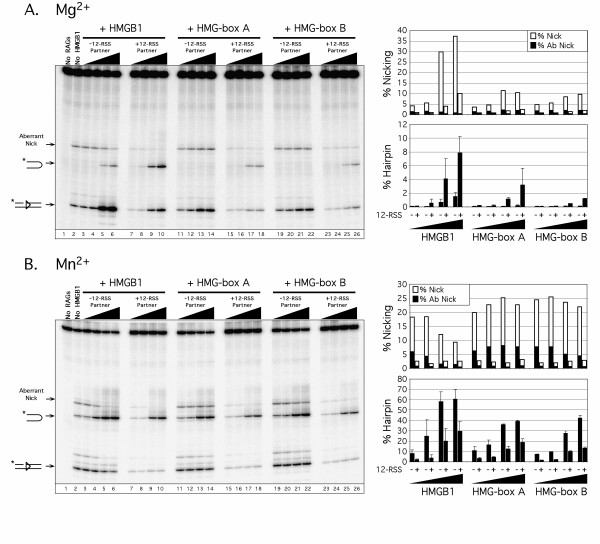
**Individual HMG-box domains stimulate RAG-mediated hairpin formation in Mn^2+^, but not Mg^2+^, in the absence of synapsis, but addition of partner RSS bypasses this impairment in Mg**^2+^. (A-B) Radiolabeled 23-RSS substrate was incubated with purified cMR1/cMR2 in an *in vitro *cleavage reaction containing of Mg^2+ ^(A) or Mn^2+ ^(B) with or without added 12-RSS in the absence or presence of increasing amounts (0.1, 10, 100 or 300 ng) of full-length HMGB1, HMG-box A, or HMG-box B at 37°C for 1 hr (Mg^2+^) or 10 min (Mn^2+^) in the combinations indicated above the gel. Reaction products were fractionated on a sequencing gel and visualized using a Storm 860 phosphorimager (left panels). The percentage of hairpin products (% Hairpin) were quantified for each reaction from at least three different gels with the average values (and standard deviations) presented in bar graph format (lower right panels). The percentage of nicks sited at the 5'-end of the heptamer (% Nick), and aberrantly introduced at position 27 (% Abnick; equivalent to nicking the substrate as a 12-RSS) is quantified from the gel shown and presented in bar graph format (open and filled bars, respectively; upper right panels). The results are representative of data obtained from at least three independent experiments.

To identify the cause of the apparent discrepancy between two previous studies regarding the ability of single HMG-box domains to stimulate RAG-mediated RSS cleavage *in vitro*, we compared RAG-mediated 23-RSS cleavage activity in the absence or presence of increasing amounts of full-length HMBG1, or individual HMG-box domains A or B (diagrammed in Fig. 1A) in an *in vitro *cleavage reaction containing Mg^2+ ^or Mn^2+ ^(diagrammed in Fig. 1B). As expected from previous studies [4,16], in buffer containing Mg^2+ ^in the absence of 12-RSS partner, the RAG proteins nick the 23-RSS (both appropriate and aberrant nicking is detected), but fail to convert nicks to DNA hairpin products (Fig. 2A, lane 2). However, under these conditions, full-length HMGB1 stimulates RAG-mediated 23-RSS nicking and hairpin formation (modestly) in a concentration-dependent manner (Fig. 2A, lanes 3–6). Aberrant nicking in the 23-RSS spacer, shown previously to occur 4 base-pairs from 3' end of the heptamer (equivalent to nicking the substrate as a 12-RSS) [18], is partially suppressed (~40%) in the presence of HMGB1. Consistent with our previous results [16], in the absence of partner RSS, HMG-box A or box B alone fails to promote RAG-mediated 23-RSS cleavage in Mg^2+^, but both HMG-box domains slightly stimulate RAG-mediated nicking in a concentration-dependent manner (Fig. 2A, lanes 11–14 and 19–22). However, no decrease in aberrant 23-RSS nicking by the RAG complex is observed. When this experiment was repeated using buffer containing Mn^2+^, we find that, in contrast to results obtained in Mg^2+^, the RAG proteins support modest 23-RSS substrate cleavage (hairpin formation) in the absence of HMGB1 (Fig. 2B, lane 2), but both full-length and individual HMG-box domains exhibit a concentration-dependent stimulation of RAG-mediated 23-RSS cleavage (Fig. 2B, lanes 3–6, 11–14, and 19–22). This result is consistent with data reported by Dai *et al *[17], suggesting that the ability of individual HMG-box domains to stimulate RAG-mediated cleavage depends on the choice of divalent metal ion used in the *in vitro *cleavage reaction. Interestingly, however, although full-length HMGB1 retains the ability to suppress aberrant 23-RSS nicking by the RAG complex in Mn^2+^, individual HMG-box domains are poorly effective in this regard. Thus, the ability of individual HMG-box domains to stimulate RAG-mediated hairpin formation is separable from their ability to suppress aberrant nicking by the RAG complex.

### Single HMG-box domains promote RAG-mediated 23-RSS cleavage under conditions favouring synapsis in both Mg^2+ ^and Mn^2+^

The finding that single HMG-box domains promote RAG-mediated 23-RSS cleavage in Mn^2+^, but not Mg^2+^, caused us to speculate that in the presence of Mn^2+^, single HMG-box domains can facilitate formation of a RAG complex bound to a 23-RSS that resembles a synaptic complex, enabling the RAG proteins to cleave the RSS in the absence of synapsis. If so, single HMG-box domains may be sufficient to stimulate RAG-mediated cleavage under conditions favouring synapsis. To test this idea, the experiments described above were repeated in the presence of a cold 12-RSS partner (Fig. 2A–B, lanes 7–10, 15–18, and 23–26). Interestingly, single HMG-box domains were found to stimulate 23-RSS cleavage in the presence, but not the absence, of 12-RSS partner, although the level of stimulation was 2-3-fold lower when compared to full-length HMGB1. This effect is lost for HMG-box B when the basic linker and acidic tail are present (data not shown). Moreover, no stimulation was observed when the composition of the partner DNA was changed from a 12-RSS to a 23-RSS (see Additional Data File 1: 12/23 dependence of RAG-mediated cleavage). It is also worth noting that aberrant 23-RSS nicking is suppressed when cold partner 12-RSS is present, regardless of the form of HMGB1 used in the cleavage reaction.

To exclude the possibility that the metal ion-dependent effects observed with polyhistidine-tagged HMGB1 on RAG-mediated RSS cleavage are not attributed to differential interactions of the polyhistidine tag with Mn^2+ ^and Mg^2+^, we compared the activity of bacterially expressed polyhistidine-tagged HMGB1 and native HMGB1 purified from calf thymus in the experiments performed in Figure 2. Although native HMGB1 was slightly more active than recombinant HMGB1 in stimulating RAG-mediated cleavage, the two forms of HMGB1 exhibited similar trends in these assays with respect to concentration- and metal ion-dependence (see Additional File 2. Recombinant and native forms of HMGB1 show similar trends in stimulating RAG-mediated 23-RSS cleavage in Mg^2+^ and Mn^2+^).  These data suggest that the distinct effects of HMGB1 on RAG-mediated cleavage in Mn^2+^ and Mg^2+^ cannot be attributed to the origin or tagging strategy of HMGB1.

### Stimulation of RAG-mediated 23-RSS cleavage by mutant and truncated forms of HMGB1 exhibits metal ion dependence

**Figure 3:**
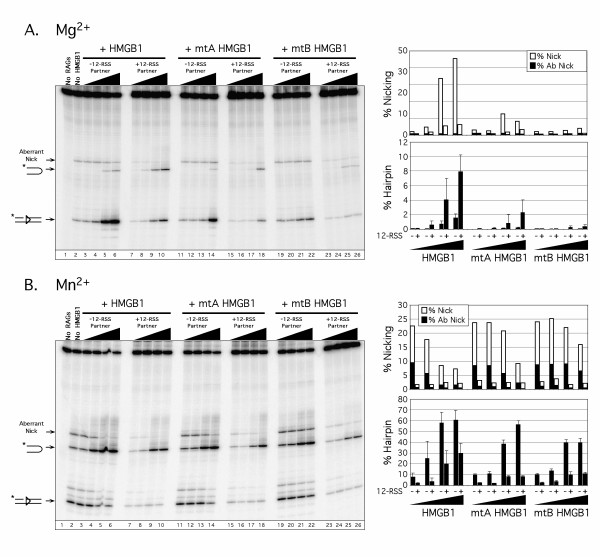
**mtA HMGB1, but not mtB HMGB1, stimulates RAG-mediated 23-RSS hairpin formation in Mg^2+^, but both proteins promote cleavage under conditions favouring synapsis in Mg^2+ ^and in reactions containing Mn**^2+^. (A-B) *In vitro *cleavage reactions were performed as in Fig. 2, except that mtA HMGB1 and mtB HMGB1 replaced HMG-box A and HMG-box B in the cleavage reactions.

The propensity of individual HMG-box domains to stimulate RAG-mediated 23-RSS cleavage in a metal ion-dependent manner caused us to ask whether other truncated and mutant forms of HMGB1, which we tested previously for their ability to stimulate RAG-mediated RSS cleavage *in vitro *[16], also exhibited metal ion-dependent effects in these assays. In the first set of experiments, we tested two mutant full-length HMGB1 proteins in which ten consecutive amino acid residues are replaced with alanine at comparable positions within either box A (starting at residue 18) or box B (starting at residue 102) (mtA and mtB, respectively; see Fig. 1A). These mutations replace key residues shown to mediate contacts to the DNA backbone (Arg^24 ^in box A and Arg^110 ^in box B) or intercalate between base steps (Phe^103 ^in box B) to promote DNA bending [19,20]; the functional importance of these residues has been confirmed by mutagenesis studies in other laboratories [21-23]. Consistent with our previous results [16], both mtA and mtB stimulated nicking in Mg^2+ ^less efficiently than full-length HMGB1 in the absence of synapsis, with mtA being slightly more effective than mtB in this regard. However, under these conditions, mtA stimulated RAG-mediated 23-RSS hairpin formation ~3-fold less efficiently than wild-type HMGB1, whereas no detectable stimulation was observed with mtB (Fig. 3A). In Mn^2+^, wild-type, mtA, and mtB HMGB1 were found to promote RAG-mediated 23-RSS nicking and hairpin formation similarly in the absence of synapsis (Fig. 3B). When these experiments were repeated in the presence of 12-RSS partner, both mtA and mtB were found to stimulate cleavage at elevated protein concentrations, but the level of stimulation relative to wild-type HMGB1 varied depending on the metal ion used in the reaction. In Mg^2+ ^at the highest protein concentration tested, mtA and mtB promoted ~4-fold and ~8-fold less cleavage, respectively, than wild-type HMGB1 (Fig. 3A). In contrast, mtA and mtB similarly stimulated hairpin formation in Mn^2+^, but only at ~30% of wild-type HMGB1 (Fig. 3B).

**Figure 4:**
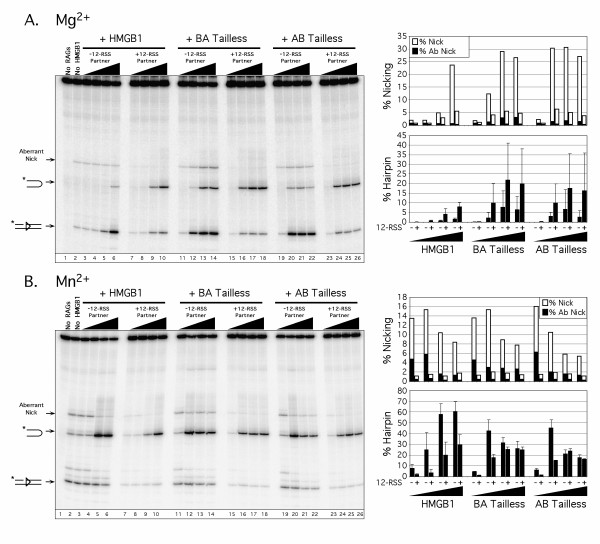
**Tailless forms of HMGB1 stimulate RAG-mediated 23-RSS cleavage at lower concentrations than full-length HMGB1, and BA Tailless exhibits a selective impairment in facilitating site-specific nicking by the RAG complex**. (A-B) *In vitro *cleavage reactions were performed as in Fig. 2, except that BA Tailless and AB Tailless replaced HMG-box A and HMG-box B in the cleavage reactions.

In the second set of experiments, we compared truncated forms of HMGB1 lacking the basic linker and acidic C-terminal tail in which box A and B were in their wild-type configuration (AB tailless) or were in reverse order (BA tailless) (see Fig. 1A). Regardless of which metal ion was tested, and whether or not 12-RSS partner was present in the reaction, both AB tailless and BA tailless were found to stimulate RAG-mediated 23-RSS cleavage at lower concentrations than wild-type HMGB1 (Fig. 4). At higher protein concentrations, both tailless forms of HMGB1 were progressively less effective at stimulating RAG-mediated nicking and hairpin formation. This outcome may be explained by the observation that removal of the acidic tail increases the affinity of tandem HMG-box proteins for DNA [12], which could result in competitive inhibition of RSS binding by the RAG complex or formation of higher-order RAG-HMGB1-RSS aggregates that are less competent for cleavage. Mobility shift assays provide experimental support for both scenarios (see below). Interestingly, AB tailless, like full-length HMGB1, partially suppresses aberrant 23-RSS nicking by the RAG complex, whereas BA tailless is unable to do so. These data suggest the orientation of HMG-box A and B relative to one another is not important for promoting RAG-mediated cleavage, but is important in guiding the correct placement of nicks.

### RAG-HMGB1-RSS complex formation is similar in Mg^2+ ^and Mn^2+^

**Figure 5:**
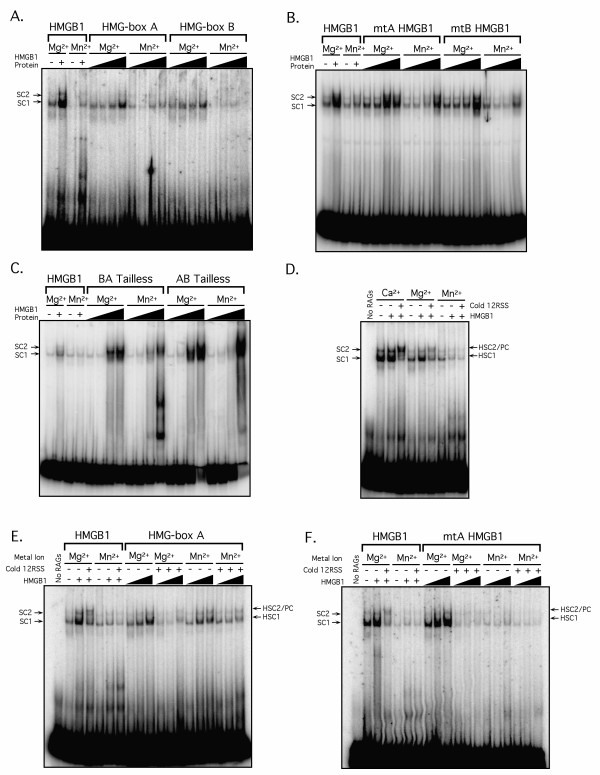
**The RAG and HMGB1 proteins exhibit similar DNA binding activity in Mg^2+ ^and Mn^2+^, but Mn^2+ ^fails to support stable RAG synaptic complex formation**. (A-C). Radiolabeled 23-RSS substrate was incubated with purified cMR1/cMR2 in binding reactions containing of Mg^2+ ^or Mn^2+^in the absence or presence of a fixed amount of full-length HMGB1 (300 ng) or increasing amounts (0.1, 10, 100 or 300 ng) of HMG-box A or box B (A), mtA or mtB HMGB1 (B), or BA Tailless or AB Tailless (C). Bound and free DNA were separated by EMSA and protein-DNA complexes visualized from dried gels using a Storm 860 phosphorimager. (D) Purified cMR1/cMR2 was incubated with a radiolabeled 23-RSS substrate in binding reactions containing Ca^2+^, Mg^2+^, or Mn^2+ ^with or without added HMGB1 (300 ng) and cold 12-RSS partner as indicated. Protein-DNA complexes were visualized as in (A-C). Assembly of the higher-order paired complex (PC) requires the presence of both HMGB1 and partner RSS, and is formed in the presence of Ca^2+ ^and Mg^2+^, but not Mn^2+^. (E-F). Radiolabeled 23-RSS substrate was incubated with purified cMR1/cMR2 in binding reactions containing Mg^2+ ^or Mn^2+ ^with or without 12-RSS partner and either a fixed amount of HMGB1 or increasing amounts of HMG-boxA (E) or mtA HMGB1 (F). Protein-DNA complexes were visualized as in (A-C).

In principle, the enhanced cleavage activity of the RAG complex in the presence of Mn^2+ ^relative to Mg^2+ ^could be attributed to metal ion-dependent differences in the DNA binding activity of the RAG and HMGB1 proteins. To test this possibility, we first compared RAG protein binding to a single 23-RSS in Mg^2+ ^or Mn^2+ ^in the absence or presence of increasing amounts of wild-type, mutant or truncated forms of HMGB1 (Fig. 5A–C). Note that because of robust RAG cleavage activity in the presence of Mn^2+ ^at 25°C (data not shown), binding reactions were assembled at 4°C. As expected from previous studies [24], two distinct protein-DNA complexes, called SC1 and SC2, are detected by EMSA when purified cMR1/cMR2 is incubated with an isolated RSS substrate in the absence of HMGB1. The more abundant SC1 complex was previously shown to contain a RAG1 dimer and monomeric RAG2, whereas the less abundant and slower migrating SC2 complex contains a RAG1/RAG2 heterotetramer [24]. In general, RAG-RSS complex formation in the absence of HMGB1 is generally slightly better in Mg^2+ ^than in Mn^2+^, in subtle contrast with previous studies showing comparable binding activity in reactions incubated at 25–30°C and subjected to glutaldehyde cross-linking [25,26]. The addition of HMGB1 to binding reactions assembled in Ca^2+ ^at 25°C was previously shown to stimulate RAG-RSS complex formation [4], and supershift both SC1 and SC2 RAG complexes, forming HSC1 and HSC2, respectively [24]. HMGB1 also stimulates RAG-RSS complex formation in both Mg^2+ ^and in Mn^2+^, with Mg^2+ ^supporting a higher level of stimulation than Mn^2+^, but supershifting of the RAG-RSS complexes is not reproducibly evident under these conditions. Our previous studies showed that the wild-type, mutant, and truncated forms of HMGB1 tested here all supershift RAG complexes assembled on a single RSS in the presence of Ca^2+ ^[16]. Under the conditions tested here, both individual HMG-box domains, and mtA and mtB HMGB1 stimulate RAG-RSS complex formation, with Mg^2+ ^supporting greater stimulation than Mn^2+ ^(Fig. 5A–B). The same trend is observed with both tailless forms of HMGB1, but supershifting of the RAG-RSS complexes at higher concentrations of these proteins is also observed (Fig. 5C). Since these data and previous studies argue that the formation of RAG-RSS and RAG-HMGB1-RSS complexes is at least equivalent, and perhaps more robust, in the presence of Mg^2+ ^relative to Mn^2+^, we conclude that the enhanced cleavage activity of the RAG and HMGB1 proteins in Mn^2+ ^relative to Mg^2+ ^cannot be attributed to metal ion-dependent differences in the DNA binding activity of these proteins.

Since HMG-box A alone and mtA HMGB1 both stimulate RAG-mediated 23-RSS cleavage in the presence, but not the absence, of 12-RSS partner, we wondered whether RAG synaptic complexes assembled with these forms of HMGB1 could be detected by EMSA. In previous studies, we and others have shown that when the RAG proteins are incubated with an appropriate pair of RSSs in the presence of full-length HMGB1, a higher-order protein-DNA complex can be detected by EMSA (the "paired complex" or "PC") that possesses intrinsically more cleavage activity than its counterpart assembled in the absence of RSS partner [24,27,28]. Normally, these complexes are assembled at 25-37°C, but rapid substrate cleavage in buffer containing Mg^2+ ^or Mn^2+ ^was observed at these temperatures, necessitating their assembly on ice. At this lower temperature, we find that full-length HMGB1 supports PC formation by the RAG proteins in the presence of Ca^2+ ^and, less efficiently, in Mg^2+^, but not in Mn^2+ ^(Fig. 5D). However, no complexes of similar mobility or exhibiting dependence on the presence of both Mg^2+ ^and partner RSS were observed in reactions containing HMG-boxA or mtA HMGB1 (Fig. 5E–F), suggesting that these forms of HMGB1 either require higher temperatures to facilitate PC assembly, or that the PCs formed are unstable toward electrophoresis.

## Discussion

Two studies aimed at identifying determinants of HMG-box proteins required to stimulate RAG-mediated cleavage *in vitro *came to conflicting conclusions about whether single HMG-box domains can promote RAG cleavage activity [16,17]. To explore whether this discrepancy can be explained by methodological differences between the two studies in the choice of protein concentration or divalent metal ion used in the *in vitro *cleavage reaction, we compared the cleavage activity of the RAG complex in Mg^2+ ^and Mn^2+ ^in the presence of increasing concentrations of various forms of HMGB1, including single HMG-box domains. Our finding that HMG-box A or box B alone can stimulate RAG-mediated cleavage in Mn^2+^, but not Mg^2+^, is consistent with both reports, and suggests the two studies can be reconciled largely based on the choice of divalent metal ion used in the *in vitro *cleavage reaction. Because HMG-box domain proteins have been implicated in promoting the activity of other nucleic acid enzymes involved in DNA replication, recombination, and repair [29-33], this study also illustrates the importance of considering the effects of metal ion composition on experimental outcomes in biochemical reactions that include HMG-box proteins.

Since the RAG proteins are known to exhibit more relaxed site specificity and more permissive cleavage activity in the absence of synapsis in Mn^2+ ^than in Mg^2+ ^[25,26], it is perhaps not surprising that this tolerant phenotype extends to the stimulation of RAG-mediated cleavage by HMG-box proteins as well. However, how Mn^2+ ^functions to promote this permissiveness, particularly with the single HMG-box domains, remains unclear. One possible clue to how this occurs is the unexpected finding that under conditions favouring synapsis, single HMG-box domains stimulate RAG-mediated 23-RSS cleavage, which otherwise does not occur in the absence of partner RSS under these conditions. This observation raises the possibility that formation of a 12/23 synaptic complex in Mg^2+ ^is associated with a conformational change in the RAG complex that stabilizes a bent DNA configuration, thereby alleviating the stringent requirement for one of the HMG-boxes which is otherwise required to facilitate RSS bending in the absence of partner RSS. We speculate that in the presence of Mn^2+^, the RAG proteins may be intrinsically more able to bend the RSS and/or stabilize a bent RSS structure, thereby enabling a single HMG-box domain to function similarly to a tandem HMG-box domain protein in promoting RAG-mediated RSS cleavage in the absence of synapsis.

## Conclusion

The tandem HMG-box domain protein HMGB1 is known to stimulate the RSS binding and cleavage activity of the RAG proteins *in vitro*. Two previous studies demonstrated that individual HMG-box domains can promote the DNA binding activity of the RAG proteins, but disagreed about whether they are also capable of stimulating RAG cleavage activity. Here we reconcile these studies by showing that the ability of single HMG-box domains to stimulate RAG-mediated RSS cleavage is metal ion-dependent. We further show that although single HMG-box domains do not stimulate RAG-mediated 23-RSS cleavage in Mg^2+^, this defect can be rescued by the addition of 12-RSS partner to promote synaptic complex formation. These results suggest synapsis leads to a conformational change in the RAG proteins that bypasses the need for one of the HMG-box domains to contact DNA.

## Methods

### RAG and HMGB1 protein purification

Truncated forms of RAG1 (amino acids 384–1040) and RAG2 (amino acids 1–387) containing an amino-terminal maltose binding protein fusion partner are diagrammed in Fig. 1A, and were coexpressed in 293 cells and purified by amylose affinity chromatography as previously described [18]. Full-length, truncated, or mutant forms of amino-terminal polyhistidine-tagged HMGB1 are diagrammed in Fig. 1A, and were expressed in *E. coli *and purified by nickel chelation chromatography followed by ion-exchange chromatography as previously described [16]. Native HMGB1 purified from calf thymus was obtained commercially (ProteinOne, Bethesda, MD).

### *In vitro *cleavage and binding assays

The 23-RSS substrate used in RAG binding and cleavage assays is 62 bp in length and is radiolabeled at the 5'-end of the top strand (diagrammed in Fig. 1B). Detailed procedures for its preparation are described elsewhere [18]. The cleavage activity of the RAG proteins was analyzed in the presence of Mg^2+ ^or Mn^2+ ^using a standard *in vitro *cleavage assay described previously [18]. Basic reactions were further supplemented with increasing amounts of various forms of HMGB1 (0.1, 10, 100, or 300 ng) without or with additional unlabeled 12-RSS or 23-RSS partner (1 pmol) as indicated. Cleavage reactions containing Mg^2+ ^were assembled at 25°C and incubated at 37°C for 1 hr, but the robust cleavage activity of the RAG complex in Mn^2+ ^without HMGB1 necessitated assembling these cleavage reactions on ice and shortening the incubation period to 10 minutes at 37°C. Cleavage reactions were terminated by adding 2 volumes of sample loading solution (95% formamide, 10 mM EDTA) and reaction products were analyzed after their separation on a sequencing gel using a Storm 860 phosphorimager running the ImageQuaNT software.

The binding of the RAG and HMGB1 proteins to the 23-RSS substrate was analyzed using an electrophoretic mobility shift assay as previously described [18], except that most binding reactions contained Mg^2+ ^or Mn^2+ ^instead of Ca^2+ ^and were assembled and incubated on ice for 10 minutes before native gel electrophoresis. Synaptic complexes were assembled under the same conditions, except that binding reactions were supplemented with cold 12-RSS partner (where indicated; 1 pmol). Protein-DNA complex formation was visualized from dried gels using the phosphorimager.

## Abbreviations

RAG1, Recombination Activating Gene-1; RAG2, Recombination Activating Gene-2; HMGB1, High Mobility Group Box 1; EMSA, electrophoretic mobility shift assay.

## Authors' contributions

PCS conceived the study, SB purified the mutant and truncated HMGB1 proteins, and ANK purified the RAG proteins and performed all experiments. PCS drafted the manuscript, which was edited into final form with input from the other authors. All authors have read and approved the final manuscript.

## Supplementary Material

Additional file 1 12/23 dependence of RAG-mediated cleavage. Gel and data analysis from an *in vitro *cleavage reaction using core RAG proteins and a labeled 23-RSS performed with or without increasing amounts of HMGB1 or HMG-box A in the absence or presence of cold partner 12-RSS or 23-RSS. Click here for file

Additional data file 2 Recombinant and native forms of HMGB1 show similar trends in stimulating RAG-mediated 23-RSS cleavage in Mg^2+ ^and Mn^2+^. Gel and data analysis from an *in vitro *cleavage reaction using core RAG proteins and a labeled 23-RSS performed with or without increasing amounts of bacterially expressed polyhistidine-tagged HMGB1 or native HMGB1 purified from calf thymus in the absence or presence of cold partner 12-RSS. Click here for file
