# Supplementary material for: HMG-box domain stimulation of RAG1/2 cleavage activity is metal ion dependent
Source: BMC Mol Biol. 2008 Apr 1;9:32. doi: 10.1186/1471-2199-9-32 (PMC2324110; doi:10.1186/1471-2199-9-32)
Supplement: Additional file 1 — 12/23 dependence of RAG-mediated cleavage. Gel and data analysis from an in vitro cleavage reaction using core RAG proteins and a labeled 23-RSS performed with or without increasing amounts of HMGB1 or HMG-box A in the absence or presence of cold partner 12-RSS or 23-RSS. [file 1471-2199-9-32-S1.pdf]

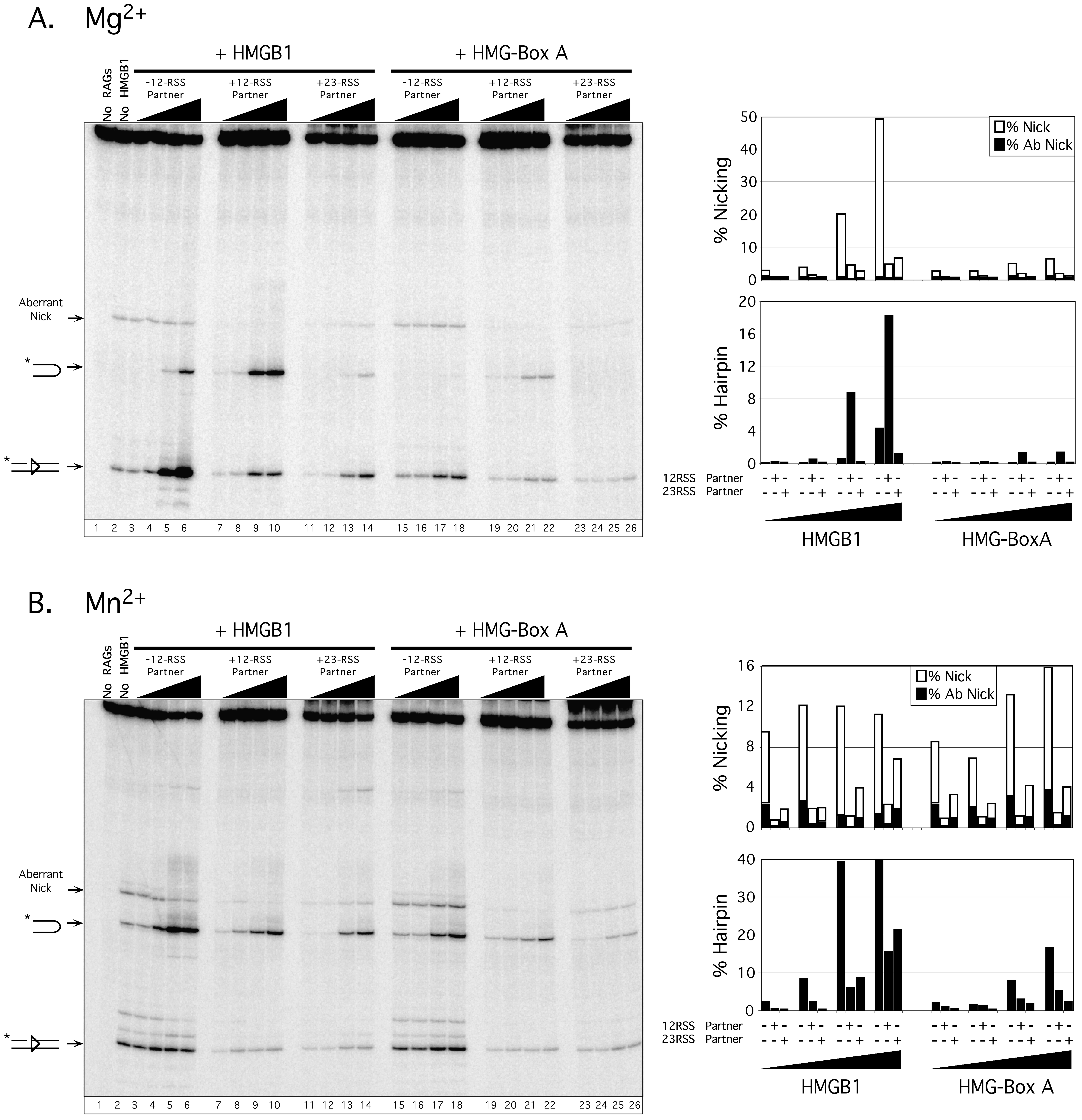

Additional Data File 1. 12/23 dependence of RAG-mediated cleavage.
Radiolabeled 23-RSS substrate was incubated with purified cMR1/cMR2 in an *in vitro* cleavage reaction containing of Mg2+ (A) or Mn2+ (B) with or without added 12-RSS or 23-RSS in the absence or presence of increasing amounts (0.1, 10, 100 or 300 ng) of full-length HMGB1 (lanes 2-14) or HMG-box A (lanes 15-26) at 37 °C for 1 hr (Mg2+) or 10 min (Mn2+) in the combinations indicated above the gel. Reaction products were fractionated on a sequencing gel and visualized using a Storm 860 phosphorimager (left panels). The percentage of hairpin products (% Hairpin) were quantified for each reaction from at least three different gels with the average values (and standard deviations) presented in bar graph format (lower right panels). The percentage of nicks sited at the 5'-end of the heptamer (% Nick), and aberrantly introduced at position 27 (% Abnick; equivalent to nicking the substrate as a 12-RSS) is quantified from the gel shown and presented in bar graph format (open and filled bars, respectively; upper right panels).
