# Supplementary material for: HMG-box domain stimulation of RAG1/2 cleavage activity is metal ion dependent
Source: BMC Mol Biol. 2008 Apr 1;9:32. doi: 10.1186/1471-2199-9-32 (PMC2324110; doi:10.1186/1471-2199-9-32)
Supplement: Additional data file 2 — Recombinant and native forms of HMGB1 show similar trends in stimulating RAG-mediated 23-RSS cleavage in Mg2+ and Mn2+. Gel and data analysis from an in vitro cleavage reaction using core RAG proteins and a labeled 23-RSS performed with or without increasing amounts of bacterially expressed polyhistidine-tagged HMGB1 or native HMGB1 purified from calf thymus in the absence or presence of cold partner 12-RSS. [file 1471-2199-9-32-S2.pdf]

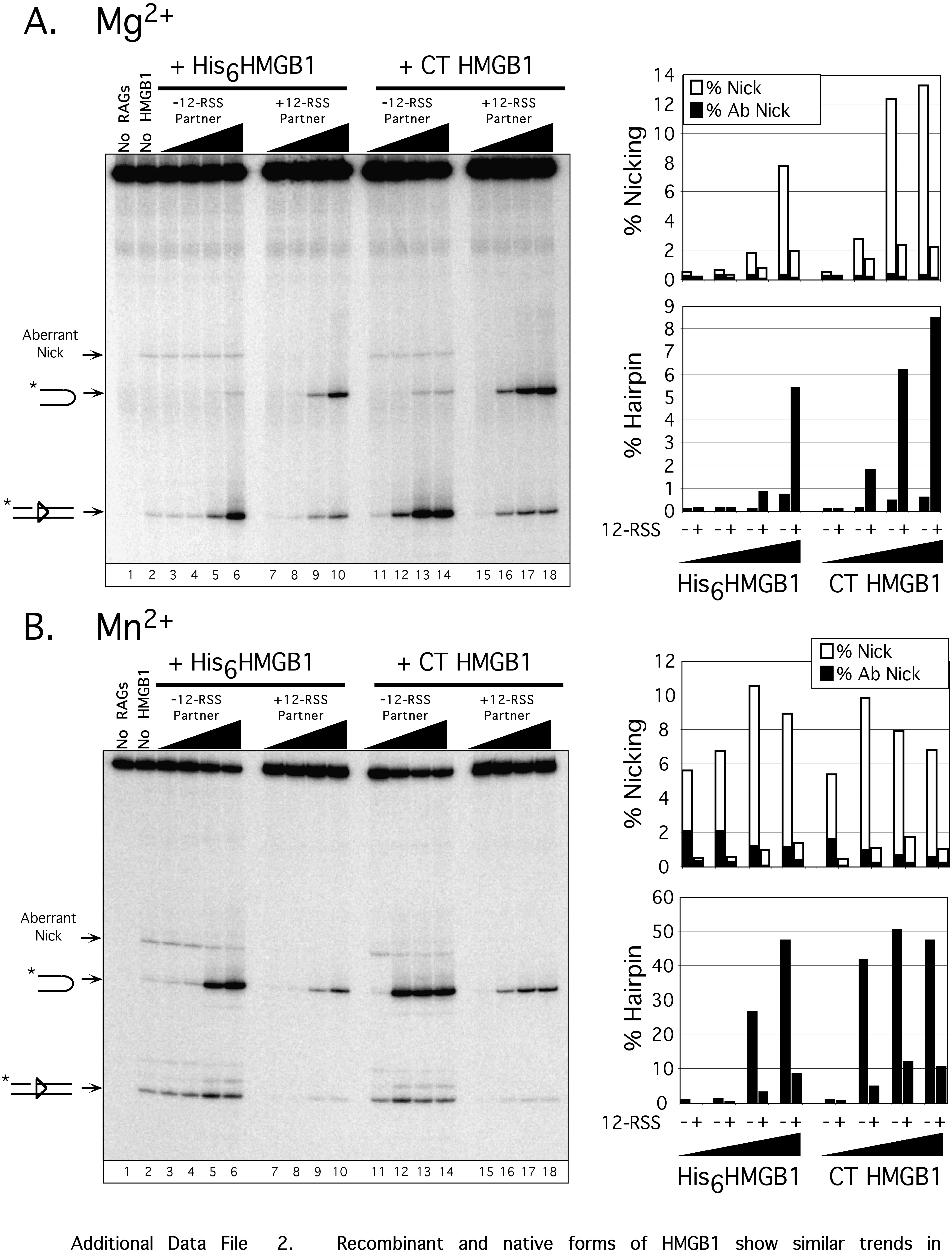

Additional Data File 2. Recombinant and native forms of HMGB1 show similar trends in stimulating RAG-mediating RSS-cleavage in Mg2+ and Mn2+.

Radiolabeled 23-RSS substrate was incubated with purified cMR1/cMR2 in an *in vitro* cleavage reaction containing of Mg2+ (A) or Mn2+ (B) with or without added 12-RSS in the absence or presence of increasing amounts (0.1, 10, 100 or 300 ng) of full-length bacterially expressed polyhistidine-tagged HMGB1 (His6HMGB1; lanes 3-10) or native HMGB1 purified from calf thymus (CT HMGB1; lanes 11-18) at 37 °C for 1 hr (Mg2+) or 10 min (Mn2+) in the combinations indicated above the gel. Reaction products were fractionated on a sequencing gel and visualized using a Storm 860 phosphorimager (left panels). The percentage of nicks sited at the 5'-end of the heptamer (% Nick), nicks aberrantly introduced at position 27 (% Abnick), and hairpin products (% Hairpin) were quantified from the gel shown and presented in bar graph format (right panels).
